# Early prediction of menopausal status after chemotherapy in women with early breast cancer in order to optimize adjuvant endocrine therapy

**DOI:** 10.1016/j.breast.2025.104562

**Published:** 2025-08-19

**Authors:** Charissa van Zwol-Janssens, Mandy M. van Rosmalen, Esther Oomen-de Hoop, Jan C. Drooger, Annemieke van der Padt-Pruijsten, Hanneke J.M. Zuetenhorst, Yvonne V. Louwers, Jenny A. Visser, Joop S.E. Laven, Agnes Jager

**Affiliations:** a Division Reproductive Endocrinology and Infertility, Department of Obstetrics and Gynecology, Erasmus MC, University Medical Center Rotterdam, the Netherlands; b Department of Medical Oncology, Erasmus MC Cancer Institute, Erasmus University MC, Rotterdam, the Netherlands; c Department of Medical Oncology, Breast Cancer Center South Holland South, Ikazia Hospital, Rotterdam, the Netherlands; d Department of Internal Medicine, Breast Cancer Center South Holland South, Maasstad Hospital, Rotterdam, the Netherlands; e Department of Medical Oncology, Franciscus Hospital, Rotterdam, the Netherlands; f Department of Internal Medicine, Erasmus MC, University Medical Center Rotterdam, Rotterdam, the Netherlands

**Keywords:** Premenopausal, Breast cancer, Chemotherapy induced amenorrhea, Ovarian function

## Abstract

**Background:**

Optimal endocrine therapy for premenopausal breast cancer patients after chemotherapy requires accurate menopausal status assessment. Current methods for determining resumption of ovarian function after chemotherapy are suboptimal. This study aims to evaluate the predictive value of pretreatment anti-Müllerian hormone (AMH) serum levels for predicting resumption of ovarian function after chemotherapy (CT).

**Methods:**

This prospective study included premenopausal women with hormone receptor-positive breast cancer undergoing CT. AMH was measured using the picoAMH assay of Anshlabs. The primary outcome was resumption of ovarian function, defined as menstrual cycle resumption or estradiol levels above 110 pmol/L within 24 months after CT.

**Results:**

Among 109 patients, pretreatment AMH was a strong predictor of resumption of ovarian function (AUC 0.86) and an optimal cut-off of 0.62 μg/L was calculated. AMH >0.62 μg/L identified women at higher risk for ovarian function resumption (sensitivity 69.9 %, specificity 88.5 %), with a false negative rate of 11.5 % and false positive rate of 30.1 %. Combining AMH and age improved predictive accuracy only slightly. No additional predictors were identified. Survival analysis confirmed that women with low pretreatment AMH (<0.62 μg/L) or older age (>40.2 years) experienced significantly less frequent and delayed ovarian function resumption.

**Conclusion:**

Pretreatment AMH is a valuable tool for predicting ovarian function resumption after chemotherapy in breast cancer patients, so that a GnRH agonist can be recommended appropriately. However, the predictive value of pretreatment AMH for permanent ovarian insufficiency is too limited to determine the postmenopausal status sufficiently accurately to switch upfront to another endocrine treatment, the aromatase inhibitors.

## Highlights

• Pretreatment AMH level predicts ovarian function resumption well.
• Pretreatment AMH level poorly predicts permanent ovarian insufficiency.
• AMH rises before ovarian function resumes.
• Age, the only other relevant factor, adds little to AMH's predictive value.
• The optimal AMH cut-off value is assay depend, assays urgently need standardization.

## 1 Introduction

Breast cancer is the most common life-threatening malignancy among women. Approximately 15 % are younger than 50 years and most of them are premenopausal at the time of diagnosis [1]. Over the years, mortality has declined partly due to improved treatment modalities. Adjuvant endocrine therapy has been instrumental in reducing mortality in patients with early hormone receptor positive (HR+) breast cancer (BC) [2]. The type of endocrine therapy prescribed is determined by menopausal status [3].

In premenopausal women, tamoxifen or aromatase inhibitor (AI) with ovarian suppression is recommended [4,5]. The combined SOFT-TEXT trials demonstrated that the addition of ovarian suppression to tamoxifen or an aromatase inhibitor significantly reduces distant (recurrence-)free survival in premenopausal women with hormone receptor-positive, HER2-negative breast cancer with a high risk of recurrence, when compared to tamoxifen alone [6,7]. Ovarian suppression is achieved through chemical castration using gonadotropin-releasing hormone (GnRH) agonists. While tamoxifen is safe for premenopausal women, AIs are not effective without co-treatment with GnRH agonists due to the continued production of estrogen in premenopausal women [8]. Misclassifying premenopausal women as postmenopausal and administering AIs without GnRH agonists is ineffective and detrimental. On the other hand, administration of a GnRH agonist in postmenopausal patients is ineffective, can cause side effects (e.g. osteoporosis) and is costly [9]. Oncologists are in clear need of practical guidance regarding the optimal use, timing, schedule, and monitoring of ovarian suppression, given the significant variation in current clinical practice [10]. Therefore, an optimal prediction of the onset of menopause after chemotherapy (CT), is crucial for optimal adjuvant endocrine therapy [11].

Among women not treated with CT, the onset of menopause can only be determined retrospectively, i.e. after 12 months of amenorrhea [12]. However, this definition is challenging for early BC patients who developed chemotherapy induced amenorrhea (CIA) given its unpredictable course. The uncertainty about resumption of ovarian function can persist up to two years after CT [13]. In clinical practice, medical oncologists use resumption of menstrual cyclicity and/or FSH and estradiol (E2) levels in the postmenopausal range to assess the resumption of ovarian function in these patients. However, these markers are not ideal because they fluctuate significantly during the menstrual cycle, and new and more accurate methods are urgently needed to determine menopausal status.

Anti-Müllerian hormone (AMH) is a useful marker for ovarian reserve and prior studies have demonstrated a link between AMH levels and the resumption of menstruation after CT [14]. However, a definitive cut-off value for predicting resumption of ovarian function remains elusive. To address this gap, our study aimed to investigate the predictive value of pretreatment AMH levels for the risk of permanent amenorrhea after CT in HR + BC patients. Moreover, we aim to identify additional predictors to use as an easily applicable, predictor for assessing resumption of ovarian function after CT in clinical practice.

## 2 Material and methods

To investigate the predictive value of pretreatment AMH levels for resumption of ovarian function after chemotherapy in premenopausal women with HR + BC in order to optimize adjuvant endocrine therapy, we performed a prospective multicenter cohort study. Patients were recruited between June 2013 and August 2022 in four centers in the area of Rotterdam: Erasmus MC, Breast Cancer Center South Holland South, location Maasstad, Ikazia, and Franciscus hospital. All patients provided written informed consent before starting study procedures. The study was registered in the Dutch Trials Registry (NL-OMON28140).

### 2.1 Patients

Premenopausal patients with early HR + BC who were planned to start treatment with (neo)adjuvant chemotherapy followed by adjuvant endocrine therapy with tamoxifen were potentially eligible to participate. Patients were considered premenopausal if they were younger than 46 years or had regular menstrual cycles and did not use any oral contraceptive pills (OCPs) or hormonal replacement therapy (HRT) before the start of (neo-) adjuvant chemotherapy. The main exclusion criteria were patients who had a bilateral oophorectomy or hysterectomy, received prior chemotherapy or pelvic radiotherapy, or used a GnRH agonist, aromatase inhibitor or hormone-containing intra-uterine devices.

### 2.2 Study design

**Fig. 1:**
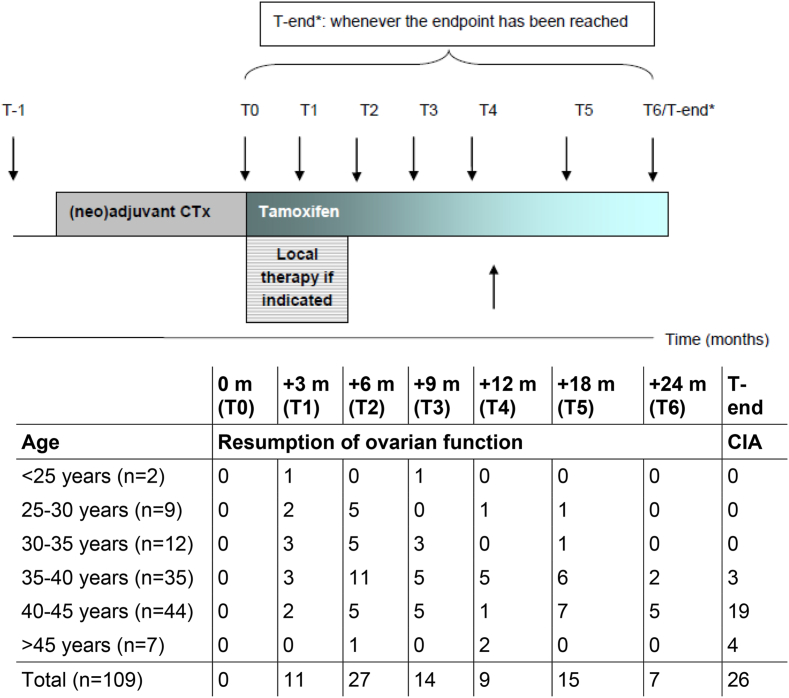
Schematic study design.

Patients were enrolled in the study at the time of breast cancer diagnosis and followed until the resumption of ovarian function or for a maximum of 24 months post-chemotherapy. Patients who did not experience any resumption of ovarian function within this 24-month ( ± 1 month) period were classified as having chemotherapy-induced amenorrhea (CIA). Blood samples were collected at baseline (pretreatment, T-1), at the end of chemotherapy (T0), and subsequently at 3, 6, 9, 12, 18, and 24 months post-chemotherapy (T1-T6) (Fig. 1).

### 2.3 Hormone measurements

Peripheral blood was collected and follicle-stimulating hormone (FSH) and estradiol (E2) levels were routinely analyzed directly in the treating center of the patients. For all time points serum was aliquoted and stored centrally at −80 °C. For AMH, all analyses were performed at the Erasmus MC with the picoAMH assay of Anshlabs® according to the manufacturer instructions. AMH values are presented in concentration of μg/L (conversion factor to pmol/L = 7.143 μg/L). The lower limit of quantification (LoQ) was 0.0221 μg/L. The analytical measurement range was 0.0067–1.00 μg/L. Stored sera were used to analyze LH and missing FSH and E2 levels.

### 2.4 Statistical analysis

The primary endpoint of the AMH study was resumption of ovarian function defined as resumption of menstrual cycle (at least more than 1 episode of vaginal bleeding, not caused by endometrial atrophy) or E2 levels in the premenopausal range (according to the local laboratory; in general above 110 pmol/l) within 24 months after chemotherapy completion. Prior to data collection, a sample size estimation was conducted based on two assumptions: (1) women diagnosed with breast cancer under the age of 40 have a 50 % probability of ovarian function resumption, while those over 40 have a 10 % probability; and (2) the study population would comprise for 25 % of women under 40 and 75 % over 40 years. Based on these assumptions, an estimated sample size of 100 women was required to achieve adequate statistical power. However, interim analysis revealed a higher than anticipated proportion of women under 40 and a greater overall incidence of resumption of ovarian function. Additionally, a significant number of participants lacked pretreatment blood samples, necessitating their exclusion from the study. Consequently, to ensure adequate representation of women older than 40 years and sufficient statistical power given the observed outcomes, the sample size was arbitrarily increased by 25 %.

Demographic and clinical characteristics were shown as means and standard deviation (SD) or medians with interquartile ranges (IQR) and compared with either parametric or non-parametric tests depending on the distribution. Receiver operating characteristic (ROC) analyses were used to assess the accuracy of prediction of resumption of ovarian function (area under the curve = AUC) and to calculate the optimal cut-off point by plotting sensitivity versus 1-specificity of pretreatment AMH, age and pretreatment AMH corrected for age. AUC values of 1.0-0.9 are classified as ‘excellent’ test quality, values of 0.9-0.8 as ‘very good’, 0.8-0.7 as ‘good’ and 0.7-0.6 as ‘satisfactory’, the rest as ‘unsatisfactory’ [15]. Furthermore, ROC analysis was used for testing the nomogram, based on age, pretreatment AMH and FSH, of the study of Xue et al. [16] in our population. We investigated the additional value of other covariates for in a nomogram with an univariate and multivariate logistic regression. To estimate the time-to-event for women with resumption of ovarian function we used Kaplan-Meier survival analysis, and the Log Rank test was performed to test equality of resumption of ovarian function distribution for AMH and age cut-offs. Finally, we evaluated the AMH, FSH and E2 levels at the different time points and compared them with a Kruskal-Wallis test. All statistical analyses were performed using IBM SPSS Statistics for Windows, version 27.

## 3 Results

**Fig. 2:**
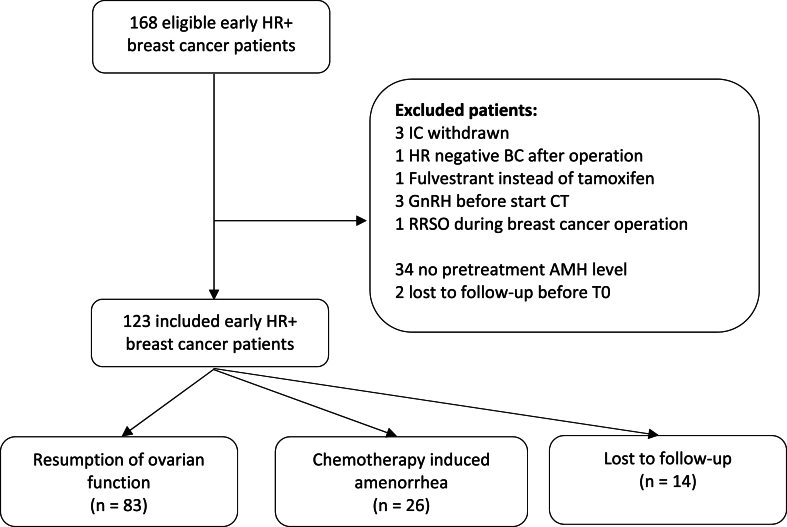
Flowchart.

A total of 168 patients were included between June 2013 and August 2022. Of these patients, 45 patients were excluded due to various reasons listed in Fig. 2. Another fourteen women were lost to follow-up before the end of the study period. Finally, 109 patients were included, 83 women (76.1 %) with resumption of ovarian function and 26 women (23.9 %) with a persisting CIA at 2 years. Fig. 1 shows the incidence of resumption of ovarian function over the follow-up period. Although younger women generally experienced more often and an earlier resumption of ovarian function, it also showed that a minority of women aged under 40 years, experienced a later resumption of ovarian function beyond 12 months. Baseline characteristics are shown in Table 1 for the total group, and the different subgroups (resumption of ovarian function, CIA and patients who were lost to follow-up). The median age of the total group was 39.2 years, for women with resumption of ovarian function this was 37.8 years and for those with CIA 42.5 years, that was statistically significantly different (p < 0.001). The other baseline characteristics were not significantly different between the subgroups. The median BMI was 24.9 kg/m^2^, the majority of the women had a Northern European ethnicity and most women received anthracyclin-taxane based chemotherapy.

**Table 1:** Baseline characteristics.

	Total premenopausal breast cancer patient (n = 123)	Resumption of ovarian function (n = 83)	Chemotherapy induced amenorrhea (n = 26)	Lost to follow-up (n = 14)	p value
**Age**, years	39.2 (35.0–42.0)	37.8 (34.4–40.9)	42.5 (40.7–44.1)	34.1 (30.5–41.7)	<0.001
**Age categories**					<0.001
<25 years	3 (2.4)	2 (2.4)	–	1 (7.1)	
25–30 years	10 (8.1)	9 (10.8)	–	1 (7.1)	
30–35 years	17 (13.8)	12 (14.5)	–	5 (35.7)	
35–40 years	36 (29.3)	32 (38.6)	3 (11.5)	1 (7.1)	
40–45 years	49 (39.8)	25 (30.1)	19 (73.1)	5 (35.7)	
>45 years	8 (6.5)	3 (3.6)	4 (15.4)	1 (7.1)	
**BMI**, kg/m^2^	24.9 (22.1–28.9)	24.9 (22.0–29.7)	25.1 (23.1–28.7)	23.9 (21.2–26.9)	0.91
**Ethnicity**					0.58
Northern European	84 (68.3)	57 (68.7)	17 (65.4)	10 (71.4)	
Mediterranean	17 (13.8)	13 (15.7)	4 (15.4)	–	
Sub-Saharan Africa	13 (10.6)	7 (8.4)	4 (15.4)	2 (14.3)	
Other/mixed	9 (7.3)	6 (7.2)	1 (3.8)	2 (14.3)	
**Gene mutation**					0.27
*BRCA1*	2 (1.6)	2 (2.4)	–	–	
*BRCA2*	2 (1.6)	2 (2.4)	–	–	
*1100delC CHEK2*	4 (3.3)	3 (3.6)	–	1 (7.1)	
Ataxia telangiectasia	1 (0.8)	1 (1.2)	–	–	
None	85 (69.1)	61 (73.5)	14 (53.8)	10 (71.4)	
**Chemotherapy regimens**					0.53
FEC	2 (1.6)	2 (2.4)	–	–	
FEC-T	64 (52.0)	46 (55.4)	12 (46.2)	6 (42.9)	
AC-T	51 (41.5)	29 (34.9)	14 (53.8)	8 (57.1)	
Other	6 (4.9)	6 (7.2)	–	–	
**Number of cyclophosphamide cycles**	3.0 (3.0–4.0)	3.0 (3.0–4.0)	4.0 (3.0–6.0)	5.0 (3.0–6.0)	0.40
**Anti-HER2 co-treatment**	25 (20.7)	18 (22.2)	6 (23.1)	1 (7.1)	0.42

Values are shown in medians with interquartile range (IQR) or in case of categorical variables in number of cases with percentages (%). BMI = body mass index; FEC = fluorouracil, epirubicin, cyclophosphamide; T = taxane; AC = doxorubicin, cyclophosphamide.

### 3.1 Prediction of resumption of ovarian function

**Fig. 3:**
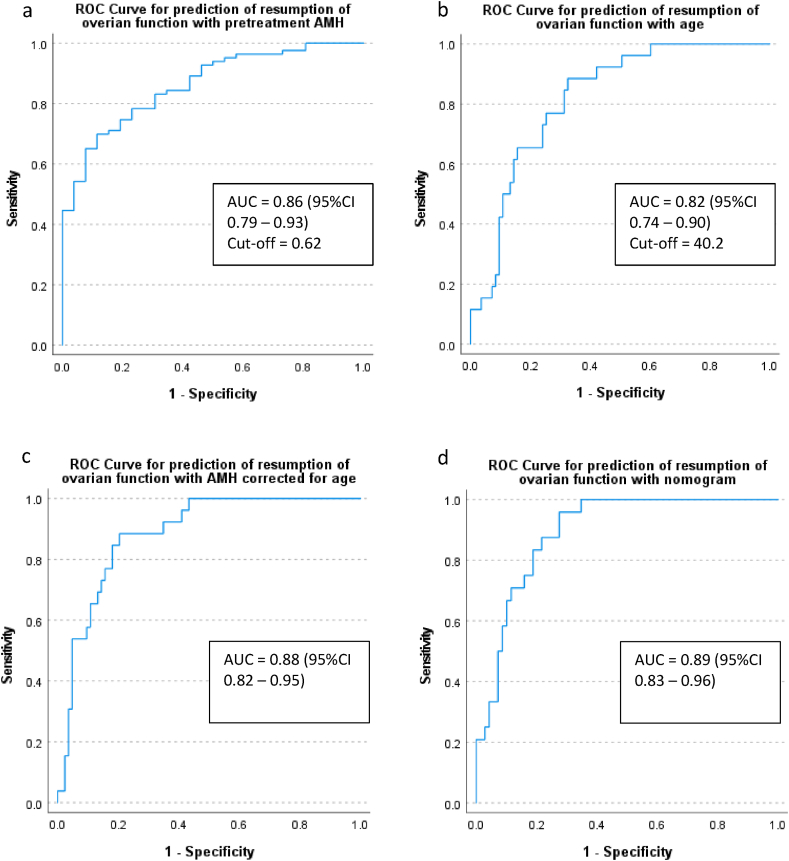
ROC curves of independent predictors ROC curves of independent predictors: a = pretreatment AMH (ovarian function n = 83; CIA n = 26); b = age (ovarian function n = 83; CIA n = 26); c = pretreatment AMH corrected for age (ovarian function n = 83; CIA n = 26); d = nomogram (ovarian function n = 69; CIA n = 24). AUC = area under the curve.

Pretreatment AMH is a very good predictor (AUC of 0.86 with (95 % confidence interval (CI) 0.79–0.93)) for resumption of ovarian function, as shown in Fig. 3a. From the ROC analysis the optimal cut-off value turned out to be 0.62 μg/L, with a sensitivity of 69.9 % and a specificity of 88.5 %. This cut-off value for AMH resulted in three (11.5 %) women who were false positive (who had an AMH >0.62 μg/L but their ovarian function *did not* resume) and 25 (30.1 %) women who were false negative (who had an AMH <0.62 μg/L but their ovarian function *did* resume). When using age as a predictor, an AUC of 0.82 (95 % CI 0.74–0.90) was calculated with an optimal cut-off value of 40.2 years (sensitivity 88.5 % and specificity 67.5 %) (Fig. 3b). This cut-off value for age resulted in three (11.5 %) women who were false positive (who had an age <40.2 year but their ovarian function *did not* resume) and 28 (33.7 %) women who were false negative (who had an age >40.2 year but their ovarian function *did* resume). In addition, we looked at a range of high sensitive cut-off of pretreatment AMH for resumption of ovarian function at 24 months (Supplemental Table 1). To maximize sensitivity (99 %) a pretreatment AMH cut-off of 0.01 μg/L was required, yielding a corresponding specificity of only 19 %, meaning that 81 % would be false positive (who had an AMH >0.01 μg/L but their ovarian function *did not* resume).

To explore if adding other covariates could improve the predictive value, we first looked at all possible predictors with an univariate logistic regression model (Table 2). Age and pretreatment AMH were significantly associated with resumption of ovarian function. Therefore, we combined pretreatment AMH with age in a multivariable logistic regression model and used this as a predictor for resumption of ovarian function. This resulted in a slightly higher AUC of 0.88 (95 % CI 0.82–0.95) compared to AMH alone. The optimal combined score had a sensitivity of 88.5 % and specificity of 79.5 % (Fig. 3c). Finally, we also calculated the total score of the nomogram of Xue et al. [16] and used this as a predictor. With an AUC of 0.89 (95 % CI 0.83–0.96), the nomogram had a similar predictive value as pretreatment AMH combined with age (Fig. 3d).

**Table 2:** Univariate and multivariate model for resumption of ovarian function.

	Univariate OR 95 % CI	Multivariate OR 95 % CI
Age	**1.44 (1.21**–**1.72)**	**1.28 (1.06**–**1.54)**
BMI	1.00 (0.92–1.09)	
Ethnicity	1.04 (0.65–1.68)	
Regimen	1.15 (0.78–1.69)	
Epirubicine doses	1.00 (1.00–1.00)	
Doxorubicine doses	1.00 (1.00–1.00)	
Cyclophosphamide doses	1.00 (1.00–1.00)	
Carboplatin doses	1.00 (1.00–1.00)	
Number of cyclophosphamide cycles	1.25 (0.90–1.75)	
AMH pretreatment	**0.12 (0.03**–**0.47)**	**0.20 (0.05**–**0.77)**
FSH pretreatment	1.05 (1.00–1.09)	

### 3.2 Rate of ovarian function resumption after chemotherapy during follow-up

**Fig. 4:**
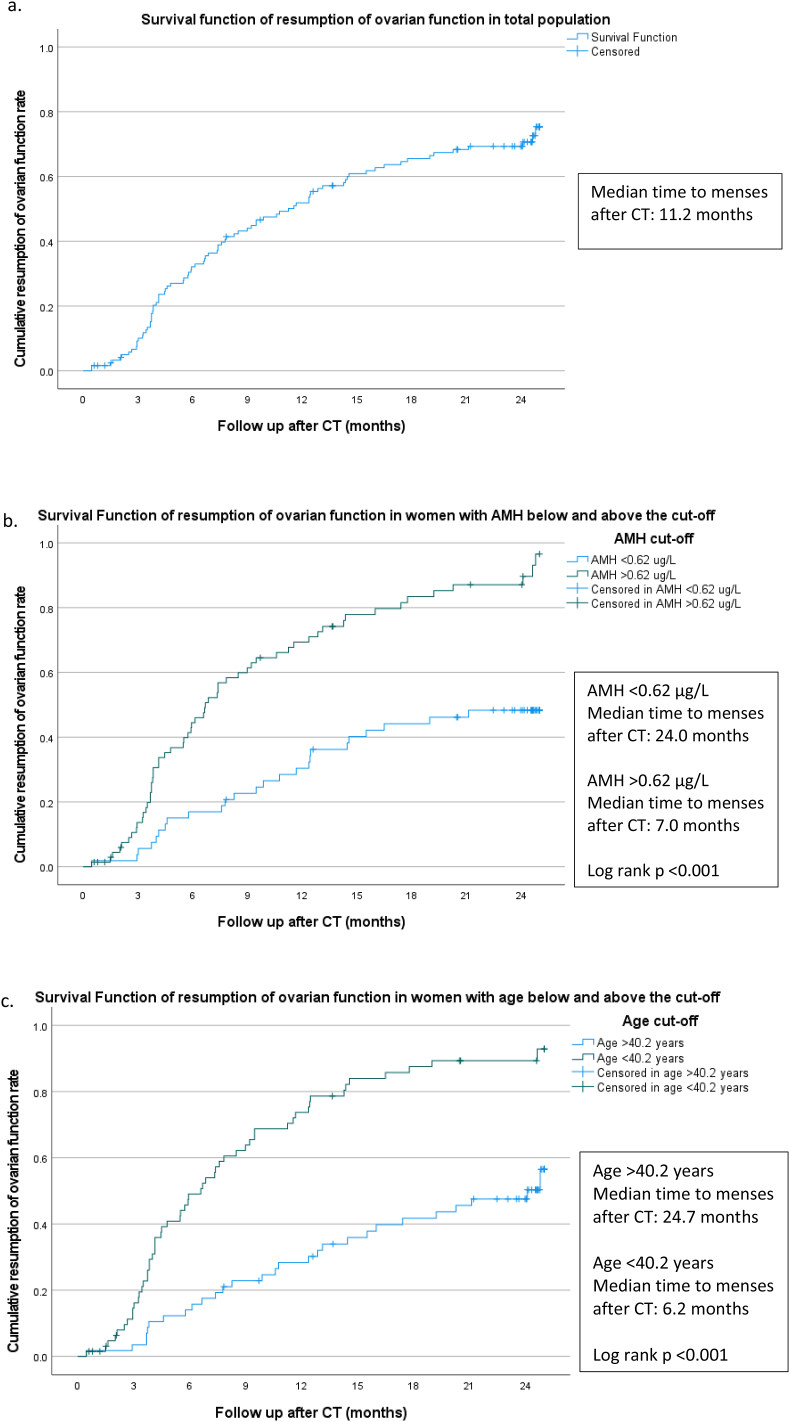
The resumption of ovarian function rate after chemotherapy over follow-up time The resumption of ovarian function rate after chemotherapy over time in the different subgroups. a = total population (n = 123); b = women with pretreatment AMH below and above the optimal cut-off value of 0.62 μg/L (below cut-off n = 53, above cut-off n = 70); c = women with pretreatment age below and above the optimal cut-off value of 40.2 years (below cut-off n = 66, above cut-off n = 57). Patients displayed with a plus mark were censored, either because they were lost to follow up or they reached the final endpoint without resumption of ovarian function.

In Fig. 4 the cumulative resumption of ovarian function rate during the follow-up after chemotherapy is shown. In the total group (n = 123) the rate of regaining ovarian function in the first 2 years is shown (Fig. 4a), women with an amenorrhea were marked with a plus sign and patients who were lost to follow-up were included in this analysis. The median time to resumption of ovarian function after CT was 11.2 months. When comparing the time to resumption of ovarian function, we found a significant difference between women with a pretreatment AMH level below (n = 53) and above (n = 70) the optimal cut-off value of 0.62 μg/L (p < 0.001) (Fig. 4b). Women with a pretreatment AMH level below the cut-off had a significantly longer time to resume ovarian function after CT (median 24.0 months) and were more likely to develop CIA (23/53 women 43.4 %), compared to women with a pretreatment AMH level above the cut-off (median 7.0 months, 3/70 women (4.3 %) developed CIA). The time to resume ovarian function was also different between women aged below or above the optimal cut-off value of 40.2 years (p < 0.001). Women with a pretreatment age above the cut-off required a significantly longer time to resume ovarian function after CT (median 24.7 months) and were more likely to develop CIA (23/57 women 40.3 %), compared to women with a pretreatment age below the cut-off (median 6.2 months, 3/66 women (4.5 %) developed CIA) (Fig. 4c).

### 3.3 Hormone levels per time point

Table 3 present the changes in AMH, FSH and estradiol levels over time in women with resumption of ovarian function and women with CIA. For time point ‘pretreatment’ and ‘T0 months’ the endpoint of the total follow-up is displayed. For the other time points (T3 months – T18 months) women in the resumption of ovarian function group, had resumption of their ovarian function before the next time point. Women in the CIA group, had no resumption of ovarian function before the next time point. Women with resumption of ovarian function had higher AMH value in the first 6 months, showing a rise just before ovarian function resumed. FSH values were not significantly lower at the timepoint before resumption of ovarian function. Estradiol levels were significantly higher in women who had resumption of ovarian function between 3 and 6 months (p = 0.007). The results of the changes over time in AMH and FSH are also graphically represented in Supplemental Figs. 1 and 2.

**Table 3:** Differences in AMH, FSH and estradiol levels between women with resumption of ovarian function and CIA per time point.

	Resumption of ovarian function∗	Chemotherapy induced amenorrhea	p value
**Pretreatment,** n = 109	83	26	
AMH, median (IQR)	1.28 (0.40–4.69)	0.12 (0.03–0.37)	<0.001
FSH, median (IQR)	5.9 (4.6–7.9)	8.9 (6.0–19.7)	0.09
Estradiol, median (IQR)	209 (104–366)	190 (109–341)	0.99
**T0 months,** n = 95	70	25	
AMH, median (IQR)	<0.001 (<0.001 - <0.001)	<0.001 (<0.001 - <0.001)	0.30
FSH, median (IQR)	81.9 (64.3–103.0)	95.4 (72.2–120.5)	0.32
Estradiol, median (IQR)	20 (<18–47)	<18 (<18–41)	0.11
**T3 months,** n = 90	17	73	
AMH, median (IQR)	0.009 (0.002–0.065)	<0.001 (<0.001 - <0.001)	<0.001
FSH, median (IQR)	55.0 (23.5–68.6)	53.4 (40.5–74.2)	0.99
Estradiol, median (IQR)	253 (75–635)	<18 (<18–56)	0.007
**T6 months,** n = 63	10	53	
AMH, median (IQR)	0.005 (<0.001–0.008)	<0.001 (<0.001 - <0.001)	<0.001
FSH, median (IQR)	41.9 (23.3–46.1)	42.4 (35.1–57.6)	0.77
Estradiol, median (IQR)	470 (66–1430)	21 (<18–54)	0.08
**T9 months,** n = 55	9	46	
AMH, median (IQR)	<0.001 (<0.001–0.008)	<0.001 (<0.001 - <0.001)	0.81
FSH, median (IQR)	30.3 (18.2–45.1)	34.5 (28.5–55.5)	0.50
Estradiol, median (IQR)	87 (<18–1140)	38 (<18–56)	0.47
**T12 months,** n = 41	10	31	
AMH, median (IQR)	<0.001 (<0.001–0.001)	<0.001 (<0.001 - <0.001)	0.49
FSH, median (IQR)	28.8 (23.7–68.2)	35.6 (25.7–50.0)	0.78
Estradiol, median (IQR)	58 (33–161)	<18 (<18–56)	0.11
**T18 months,** n = 12	2	10	
AMH, median (IQR)	<0.001	<0.001 (<0.001 - <0.001)	1.00
FSH, median (IQR)	27.4	37.5 (21.3–47.2)	0.46
Estradiol, median (IQR)	892	21 (<18–60)	0.46

AMH levels in micrograms/liter (μg/L), FSH in international unit/liter (IU/L) and estradiol in picomolar/liter (pmol/L) at the different time points. Levels are displayed in two groups, women with resumption of ovarian function and chemotherapy induced amenorrhea (CIA). ∗For time point pretreatment and T0 months the endpoint of the total follow-up is displayed, for the other time points women in the resumption of ovarian function group their ovarian function resumed before the next time point and women in the CIA group had no resumption of ovarian function before the next time point.

## 4 Discussion

In our large prospective study, we have shown that in premenopausal breast cancer patients, over 76 % had resumption of ovarian function with currently used chemotherapeutic regimens within 24 months after chemotherapy completion. This resumption can be very well predicted by a pretreatment AMH level using the Anshlabs picoAMH assay. A cut-off value of 0.62 μg/L had the highest discriminatory power, and this cut-off value could also identify patients who have a significantly shorter time to resumption of ovarian function after chemotherapy. Finally, we found that AMH levels after chemotherapy rose just before ovarian function resumed.

Other studies have reported a wide ranges in the percentages for resumption of ovarian function, varying between 23 % and 92 % within 2 years of follow-up [[16], [17], [18], [19], [20], [21], [22]]. The age differences among participants likely account for this variation. Our results regarding AMH are in line with previous research, reporting a good to excellent predictive value of pretreatment AMH for resumption of ovarian function after chemotherapy (AUC ranging from 0.77 to 0.91) [16,[21], [22], [23], [24]]. While prior studies have proposed various AMH cut-off values for this purpose based on different AMH assays [16,[22], [23], [24]], our study is the first to establish a cut-off for the Anshlabs picoAMH assay capable of detecting even the lowest levels of AMH. Due to the lack of an international AMH standards and the dependence of interassay correlation on serum AMH concentration [25], each AMH assay should ideally have its own cut-off value. Importantly, our findings reveal that this cut-off can effectively identify patients with significantly longer time to resumption of ovarian function. Therefore, a cut-off can be a valuable tool for clinicians in identifying patients with (an earlier) resumption of ovarian function. This information can inform treatment decisions, such as the type of endocrine therapy and, more importantly, whether or not a GnRH agonist should be added before ovarian function has resumed.

Although pretreatment AMH demonstrates strong discriminatory power in predicting ovarian function resumption, it is important to note that the cut-off value has a low positive predictive value for permanent CIA, highlighting a potential for misclassification. For optimal adjuvant endocrine therapy, accurately identifying patients with permanent CIA is crucial. Postmenopausal women are typically candidates for AIs, whereas these agents can be detrimental to premenopausal women if used without GnRH agonist. Thus, AMH effectively identifies women with ovarian function resumption, its predictive power for CIA decreases at lower AMH levels. In our study, approximately one in three women had a pre-chemotherapy AMH level of <0.62 μg/L but their ovarian function did resume. Given the observed high percentage of ovarian function resumption in premenopausal women of over 75 % together with the high negative predictive value of the AMH cut-off value for permanent CIA, we propose treating all women with an AMH level above 0.62 μg/L with tamoxifen or an AI combined with a GnRH agonist. Conversely, women with an AMH value below this cut-off could be treated with tamoxifen (or even an AI), along with regular check-ups for the resumption of ovarian function (using estradiol levels). It is prudent to anticipate the return of menstruation, even after 2 years, in women with an AMH level significantly below the 90th percentile. After all, it turned out to be that three women regained ovarian function in the 24th month, two of whom had high pretreatment AMH levels. A potential explanation for these unexpected late recoveries could be the presence of polycystic ovary syndrome (PCOS) in these individuals, because they have AMH values far above the 90th percentile for their age suggesting polycystic ovary morphology (PCOM) [26]. Although all women had a regular cycle prior to chemotherapy, these women could still have PCOS. Consequently, the prolonged absence of menstruation post-chemotherapy before resumption might be more attributable to the natural fluctuations of PCOS rather than solely to the effects of chemotherapy.

Two previous studies have examined the trajectory of AMH levels after chemotherapy, both demonstrating that AMH after CT is significantly higher in women who had resumption of ovarian function compared to those who experience CIA [20,22]. In our study, we looked even deeper into this topic and found that among women who had resumption of ovarian function, AMH levels rose just before ovarian function resumed. This finding can be helpful during follow-up in women where there is uncertainty regarding the resumption of ovarian function, for example by routinely monitoring AMH level instead of E2 and FSH levels in these women.

To optimize the predictive value of our model, we explored the potential contribution of additional factors. While AMH demonstrated strong predictive capabilities, incorporating age into the model provided only marginal improvements, suggesting that AMH is a more accurate indicator of pretreatment ovarian aging than chronological age. This finding is in line with previous research that has consistently shown that AMH is more closely correlated with the functional status of the ovaries than calendar age [27]. Our results contribute to the growing body of evidence that AMH is a valuable biomarker for ovarian reserve, not only in the context of fertility but also in other malignancies such as breast cancer. However, it is important to note that the relationship between AMH and breast cancer is complex, as the disease itself can also influence AMH levels. It has been observed that AMH is often lower at the time of a cancer diagnosis [28]. We also investigated the predictive value of other potential factors including BMI, ethnicity, treatment regimen, dosages, and number of cycles, but none of these variables significantly improved the model. Additionally, we compared the performance of our model to the nomogram developed by Xue et al. [16], finding similar AUC values. To assess the potential for early prediction of ovarian function recovery, we analyzed changes in AMH and FSH levels over time post-chemotherapy. While we observed a consistent rise in AMH levels preceding the resumption of ovarian function, the timing of this increase varied substantially among patients. Consequently, we were unable to identify a specific time point for AMH measurement that could reliably predict ovarian function recovery.

Our study benefits from a relatively large sample size, prospective design, and multicenter collaboration, allowing for a robust assessment of ovarian function recovery over a two-year follow-up period. This design enabled us to capture actual resumption of ovarian function data. However, despite including 113 patients from various Dutch centers, we observed limited heterogeneity in covariates, potentially hindering the identification of additional predictors. Furthermore, a significant amount of missing data in follow-up hormone values, due to factors such as cycle timing and sample loss, impacted our ability to fully explore the dynamic changes in hormonal profiles during the two year follow-up period. An other important note is that the cut-off value of 0.62 μg/L is specific to the Anshlabs picoAMH assay. The absence of an international standard for AMH underscores the need for further standardization and validation of other AMH assays to ensure consistent and reliable results across different laboratories.

In conclusion, our study demonstrates that pretreatment AMH is a strong predictor of ovarian function resumption following chemotherapy in premenopausal breast cancer patients. The identified AMH cut-off value using the Anshlabs picoAMH assay offers a valuable tool for clinicians in identifying premenopausal women who would benefit from adding GnRH agonists to tamoxifen. While AMH provides significant predictive power, its limitations in accurately predicting permanent ovarian insufficiency highlights the need for further research to refine its clinical utility. Future studies should focus on identifying additional predictive factors, standardizing AMH assays, and exploring the dynamic changes in hormonal profiles following chemotherapy to improve the prediction of ovarian function recovery and inform optimal treatment decisions.

## CRediT authorship contribution statement

**Charissa van Zwol-Janssens:** Writing – original draft, Methodology, Formal analysis. **Mandy M. van Rosmalen:** Writing – review & editing, Writing – original draft, Resources, Methodology, Formal analysis. **Esther Oomen-de Hoop:** Writing – review & editing, Methodology, Formal analysis. **Jan C. Drooger:** Writing – review & editing, Resources. **Annemieke van der Padt-Pruijsten:** Writing – review & editing, Resources. **Hanneke J.M. Zuetenhorst:** Writing – review & editing, Resources. **Yvonne V. Louwers:** Writing – review & editing, Supervision, Methodology, Conceptualization. **Jenny A. Visser:** Writing – review & editing, Supervision, Methodology. **Joop S.E. Laven:** Writing – review & editing, Supervision, Methodology, Conceptualization. **Agnes Jager:** Writing – review & editing, Supervision, Resources, Methodology, Conceptualization.

## Ethical approval

All patients provided written informed consent before starting study procedures. The study was registered in the Dutch Trials Registry (NL-OMON28140).

## Funding

This work was supported by the 10.13039/501100004622Koningin Wilhelmina Fonds (10.13039/501100004622KWF) [grant number EMCR 2016-8179] and Pink Ribbon [project number 2011-WO38. C87].

## Declaration of competing interest

The authors declare the following financial interests/personal relationships which may be considered as potential competing interests:M.M. van Rosmalen reports a relationship with Astrazeneca that includes: consulting or advisory. Y.V. Louwers reports a relationship with Erasmus MC that includes: funding grants. Y.V. Louwers reports a relationship with Ferring that includes: speaking and lecture fees. Y.V. Louwers reports a relationship with Merck & Co Inc that includes: speaking and lecture fees. J.A. Visser reports a relationship with Erasmus MC that includes: non-financial support. J.S.E. Laven reports a relationship with Ansh Labs LLC that includes: funding grants. J.S.E. Laven reports a relationship with Ferring that includes: funding grants. J.S.E. Laven reports a relationship with Merck & Co Inc that includes: funding grants. J.S.E. Laven reports a relationship with National Institutes of Health that includes: funding grants. J.S.E. Laven reports a relationship with Ansh Labs LLC that includes: consulting or advisory. J.S.E. Laven reports a relationship with Ferring that includes: consulting or advisory. J.S.E. Laven reports a relationship with Gedeon Richter Plc that includes: consulting or advisory. J.S.E. Laven reports a relationship with Roche Diagnostics that includes: consulting or advisory. J.S.E. Laven reports a relationship with Titus Health care that includes: consulting or advisory. If there are other authors, they declare that they have no known competing financial interests or personal relationships that could have appeared to influence the work reported in this paper.
