# Supplementary material for: Early prediction of menopausal status after chemotherapy in women with early breast cancer in order to optimize adjuvant endocrine therapy
Source: Breast. 2025 Aug 19;83:104562. doi: 10.1016/j.breast.2025.104562 (PMC12397871; doi:10.1016/j.breast.2025.104562)
Supplement: Multimedia component 1 [file mmc1.docx]

*Supplemental table 1*. A range of high sensitive pretreatment AMH cut-offs for resumption of ovarian function at 24 months

| Sensitivity | 99% | 98% | 95% | 90% | 85% |
| --- | --- | --- | --- | --- | --- |
| Cut-off (ng/mL) | 0.01 | 0.03 | 0.11 | 0.14 | 0.17 |
| Sensitivity (%) | 98.8 | 97.6 | 95.2 | 90.4 | 85.5 |
| Specificity (%) | 19.2 | 26.9 | 46.2 | 53.8 | 57.7 |
| False positive rate (%) | 83.4 | 77.8 | 75.1 | 63.7 | 55.5 |

We listed a range of relevant sensitivities (99%, 98%, 95%, 90% and 85%) and looked up the closest sensitivity in the coordinates of the ROC curve.


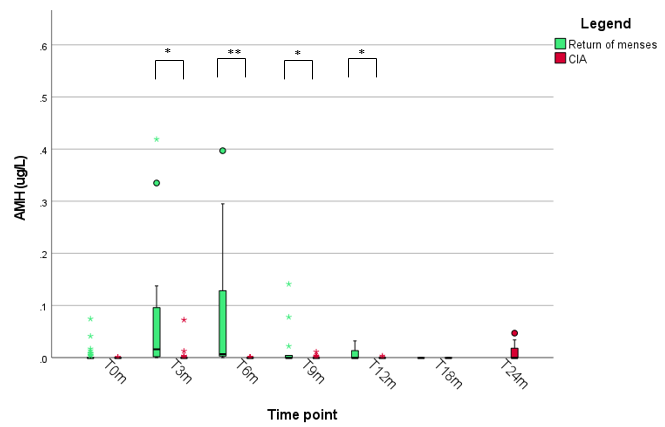

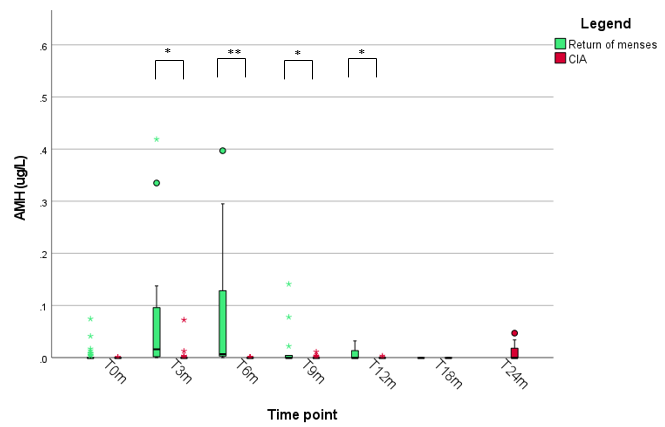
*Supplemental figure 1*. Differences in AMH levels between women with resumption of ovarian function and CIA per time point

**


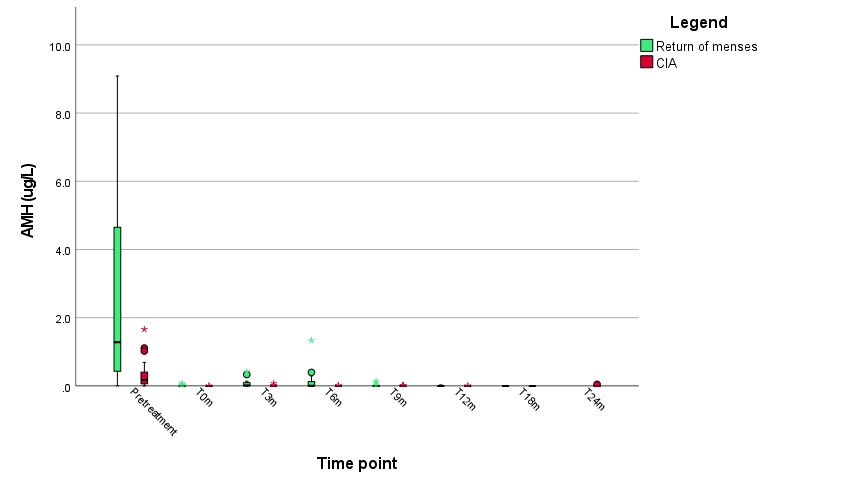


AMH levels in micrograms / liter (µg/L) at the different time points. Levels are displayed in two groups, women with resumption of ovarian function and chemotherapy induced amenorrhea (CIA). For time point pretreatment and T0 months the endpoint of the total follow-up is displayed, for the other time points women in the resumption of ovarian function group their ovarian function resumed before the next time point and women in the CIA group had no resumption of ovarian function before the next time point. * p < 0.05; ** p < 0.001.

*Supplemental figure 2*. Differences in FSH levels between women with resumption of ovarian function and CIA per time point


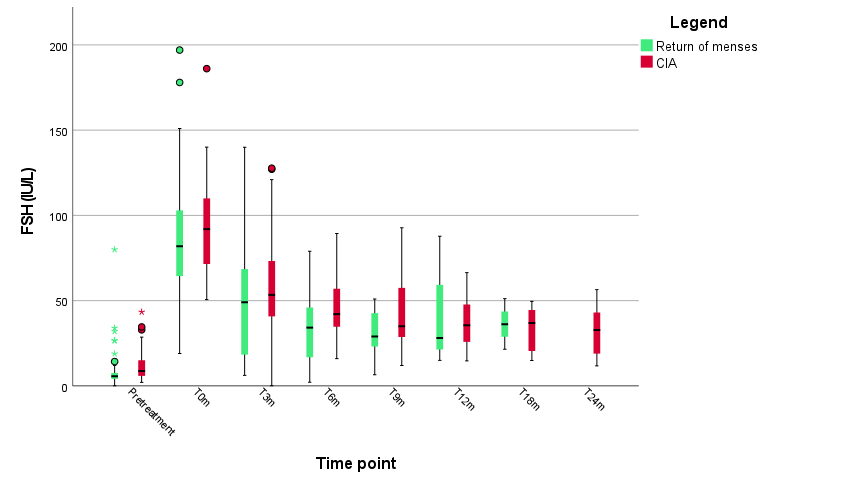


*

*

FSH levels in international unit / liter (IU/L) at the different time points. Levels are displayed in two groups, women with resumption of ovarian function and chemotherapy induced amenorrhea (CIA). For time point pretreatment and T0 months the endpoint of the total follow-up is displayed, for the other time points women in the resumption of ovarian function group their ovarian function resumed before the next time point and women in the CIA group had no resumption of ovarian function before the next time point. * p < 0.05.
